# *TP73-AS1* is induced by YY1 during TMZ treatment and highly expressed in the aging brain

**DOI:** 10.18632/aging.203182

**Published:** 2021-06-11

**Authors:** Gal Mazor, Dmitri Smirnov, Hila Ben David, Ekaterina Khrameeva, Debra Toiber, Barak Rotblat

**Affiliations:** 1 Department of Life Sciences, Ben-Gurion University of the Negev, Beer Sheva 8410501, Israel; 2 Center of Life Sciences, Skolkovo Institute of Science and Technology, Moscow 121205, Russia; 3 The Zlotowski Center for Neuroscience, Ben-Gurion University of the Negev, Beer Sheva 8410501, Israel; 4 The National Institute for Biotechnology in the Negev, Beer Sheva 8410501, Israel

**Keywords:** glioblastoma, long noncoding RNA

## Abstract

Aging is a factor associated with poor prognosis in glioblastoma (GBM). It is therefore important to understand the molecular features of aging contributing to GBM morbidity. *TP73-AS1* is a long noncoding RNA (lncRNA) over expressed in GBM tumors shown to promote resistance to the chemotherapeutic temozolomide (TMZ), and tumor aggressiveness. How the expression of *TP73-AS1* is regulated is not known, nor is it known if its expression is associated with aging. By analyzing transcriptional data obtained from natural and pathological aging brain, we found that the expression of *TP73-AS1* is high in pathological and naturally aging brains. YY1 physically associates with the promoter of *TP73-AS1* and we found that along with *TP73-AS1*, *YY1* is induced by TMZ. We found that the *TP73-AS1* promoter is activated by TMZ, and by YY1 over expression. Using CRISPRi to deplete YY1, we found that YY1 promotes up regulation of *TP73-AS1* and the activation of its promoter during TMZ treatment. In addition, we identified two putative YY1 binding sites within the *TP73-AS1* promoter, and used mutagenesis to find that they are essential for TMZ mediated promoter activation. Together, our data positions YY1 as an important *TP73-AS1* regulator, demonstrating that *TP73-AS1* is expressed in the natural and pathological aging brain, including during neurodegeneration and cancer. Our findings advance our understanding of *TP73-AS1* expression, bringing forth a new link between TMZ resistance and aging, both of which contribute to GBM morbidity.

## INTRODUCTION

Glioblastoma multiform (GBM) is a cancer of the brain with a dismal outcome and a five-year survival rate of < 10% [1]. Its location in the brain, recurrence, and tendency to infiltrate areas adjacent to the primary tumor are some of the major features contributing to its aggressiveness. Current treatments rely on surgical resection, radio therapy and temozolomide (TMZ) administration [2], which extends patient survival. However, therapy resistance in tumor cells is a major limiting factor in therapeutic success [3, 4]. It is therefore important to advance our understanding of resistance mechanisms for the development of new much needed therapies.

Aging contributes to cancer [5, 6] and is a factor associated with poor survival in GBM animal models [7] and patients [8, 9]. In particular, epi-genetic changes affecting gene expression strongly correlated with age in low grade glioma [10]. The molecular mechanisms linking aging and GBM are still vague and were proposed to be related to the gain of function of tumor associated fibroblasts [11] and functional decline of the immune system [7]. Nevertheless, the molecular details linking aging to GBM aggressiveness are still vague.

Long noncoding RNA (lncRNA) are regulatory RNA molecules known to play important roles in cancer such as promoting resistance to therapy [12, 13]. The lncRNA *TP73-AS1* is a gene neighbor of the transcription factor (TF) p73, a member of the p53 TF family [14] known to play important roles in aging [15–17], cancer [18–23] and brain development [24–27] by regulating gene expression at the transcriptional and translational levels [28, 29]. LncRNA function by diverse mechanisms including by regulating gene expression in *cis* [30] however, *TP73-AS1* does not regulate *p73* in GBM stem cells [31] and to best of our knowledge was not found to regulate *p73* in other biological scenarios.

*TP73-AS1* is a negative prognostic factor in several tumor types [32]. In glioma, the expression of *TP73-AS1* was shown to be associated with poor patient outcome and, importantly, with aging [33]. In GBM cancer stem cells, *TP73-AS1* was found to promote TMZ resistance by facilitating the expression of the TMZ detoxifying enzyme, *ALDH1A1* [31]. In accordance, *TP73-AS1* is clinically relevant in GBM and its high expression in GBM tumors is associated with poor patient outcome. Furthermore, *TP73-AS1* is highly expressed in the more aggressive IDH WT and EGFR amplified GBM tumors [31]. Interestingly, *TP73-AS1* is relevant and functional in other brain tumors including medulloblastoma [34] and astrocytoma [35, 36]. Nevertheless, how the expression of *TP73-AS1* is regulated in the context of TMZ treatment is not known, nor is it known if the expression of *TP73-AS1* is associated with aging in brain.

Here we found that *TP73-AS1* is highly expressed in the aging brain, and under pathological conditions, its expression is increased. We discovered that the TF Yin Yang 1 (YY1) regulates *TP73-AS1* expression. YY1 was known to contribute to TMZ resistance by promoting the expression of DNA repair genes [37], and to be a part of the transcriptional program of the aging brain [38]. Importantly, YY1 directly activates the transcription of *TP73-AS1* upon TMZ treatment. Together, these findings bring forth a new mode of regulation implicated in TMZ resistance in GBM and an intriguing link between aging and GBM.

## MATERIALS AND METHODS

### RNA extraction, cDNA synthesis, and quantitative reverse transcription polymerase chain reaction (qRT-PCR)

RNA from cultured cells was purified using the NUCLEOSPIN RNA PLUS (Macherey-Nagel). From a starting amount of 100–200 ng of RNA, cDNA was generated using the cDNA synthesis kit (E6300S, BioLabs). Briefly, 20 μl of the cDNA synthesis reaction was subjected to the following conditions: 5 min at 25° C, 60 min at 42° C, and 5 min at 80° C. The qPCR conditions were as follows: initialization step at 95° C for 10 min, 46 cycles of the denaturation step at 60° C for 30 s, annealing step at 60° C for 30 s, and elongation step at 72° C for 1 s, followed by the final elongation at 40° C for 10 min to ensure that any remaining single-stranded DNA is fully extended. Forward and reverse primers were designed to span different exons whenever possible. All primers were purchased from Integrated DNA Technologies (IDT). qRT-PCR was performed using an iCycler (Bio-Rad Laboratories) with a threshold cycle number determined with the use of iCycler software version. Reactions were performed in triplicate and threshold cycle numbers were averaged. The results were normalized to L32.

### RT-PCR primers used in this study

TP73-AS1: probe #29, primer1 ctccggacactgtgttttctc, primer2 tcttttaaggcggccatatc;

L32: probe #33, primer1 gcacactgactacagccttga, primer2 taccc aggtttggaggtgtg;

YY1: probe #79: primer1 ttggagagaactcacctcctg, primer2 gccgagttatccctgaacat;

ZEB1: probe #68: primer1 gccaacagaccagacagtgtt, primer2 tttcttgcccttcctttctg;s.

### Plasmids

psPAX2 was a gift from Didier Trono (Addgene plasmid # 12260). pMD2.G was a gift from Didier Trono (Addgene plasmid # 12259). pHAGE TRE dCas9-KRAB was a gift from Rene Maehr and Scot Wolfe (Addgene plasmid # 50917). pLKO.1-puro U6 sgRNA BfuAI large stuffer was a gift from Scot Wolfe (Addgene plasmid # 52628). Rennila and luciferase (pGL 4.23) vectors were a kind gift from Gabriel Leprivier. The two parts of the promoter (824BP and 1124BP) were cloned into the Firefly luciferase using TEDA method [39] between XhoI and HindIII restriction enzyme sites. The mutated YY1 binding site promoter was cloned using standard site directed mutagenesis. YY1 over expression vector was described in [38]. We used pLKO.1-puro U6 sgRNA BfuAI large stuffer. pLKO.1-puro U6 sgRNA BfuAI large stuffer was a gift from Scot Wolfe (Addgene plasmid # 52628). The primer sequences used were as follows:

scramble/control: ACCGCGCCAAACGTGCCCTGACGG

YY1 g#1: GGAGACCATCGAGACCACAG;

YY1 g#2: CGACACCCTCTACATCGCCA.

### Generation of stable cell lines for gene knockdown

Lentiviruses were generated using our standard protocol and LentiX cells. Cells were grown in DMEM, 10% FBS, and transfected using CalFectin (Signagen) and plasmids at a ratio of 1:2:3 (PAX2; pMD2.G; transfer vector). The media was changed after 24 h and the viruses were collected after 48 h. The collected viruses were stored at −80° C. G7 and SHSY5Y cells were grown to 80% confluence on 6-cm dishes, then infected with a vector ratio of 1:10 (cas9, gRNA), and left for 24 h in a 37° C incubator. Following infection, the lentivirus containing medium was removed and replaced by fresh medium and incubated at 37° C with the selection antibiotic using puromycin (1 μg/ml) and G418 (1500 μg/ml).

### CRIPSRi

The depletion of the YY1 was done using the CRISPRi method combined with viral infection. gRNA were designed to target the promoter region of the YY1 gene. Knockdown induction was done using doxycycline at a concentration of 2 μg/ml (Sigma-Aldrich, D3072). Gene knockdown was measured using qRT-PCR.

### TMZ treatment

YY1 KD was induced by addition of doxycycline for 10 days. Luciferase Activity of the different TP73-AS1 promoter parts was measured by luciferase reporter gene assay (Promega, E1500) in SHSY5Y and HEK293 cell lines. The SHSY5Y cells were transient transfected (Lipojet, BioConsult SL100468) with the reporter and control plasmids, and were cultured in DMEM medium with 10% fetal bovine serum. 24h later, some wells were treated with DMSO and others with TMZ (750uM) for 2 days. Luciferase activity was measured and normalized by measuring co-transfected Renilla plasmid using the Dual-Luciferase® Reporter Assay System, Promega.

### Western blot

Western blots were performed as in [40]. In short, lysates were mixed with sample loading buffer 5X (250 mM Tris·HCl pH 6.8; 10% SDS; 30% Glycerol; 10 mM DTT; 0.05% (w/v) Bromophenol Blue), followed by 10 minutes incubation at 96° C and spin down. Samples were then loaded to SDS-PAGE followed by transferring to nitrocellulose membrane. Membranes were then blocked by soaking in milk (5% skim milk; Tris buffer saline x1 contains 0.05% TWEEN20 (TBST)) for 1 hour. Membranes were then covered with primary antibody against *YY1* solution (abcam ab109237) for 1 hour in room temperature or overnight in 4° C, followed by washing with TBST and incubation with secondary antibodies (Cell signaling technology,7074S) for 1 hour in room temperature.

### Statistical analysis

Salomon (GSE5281) [41], Cotman (GSE48350) [42], GTEx [43] and ICGC GBM-US [44] datasets were used for analysis. Wilcoxon rank test was used to determine statistical significance between groups. Statistical significance was set to 0.05.

## RESULTS

### *TP73-AS1* is highly expressed in the aging brain

**Figure 1:**
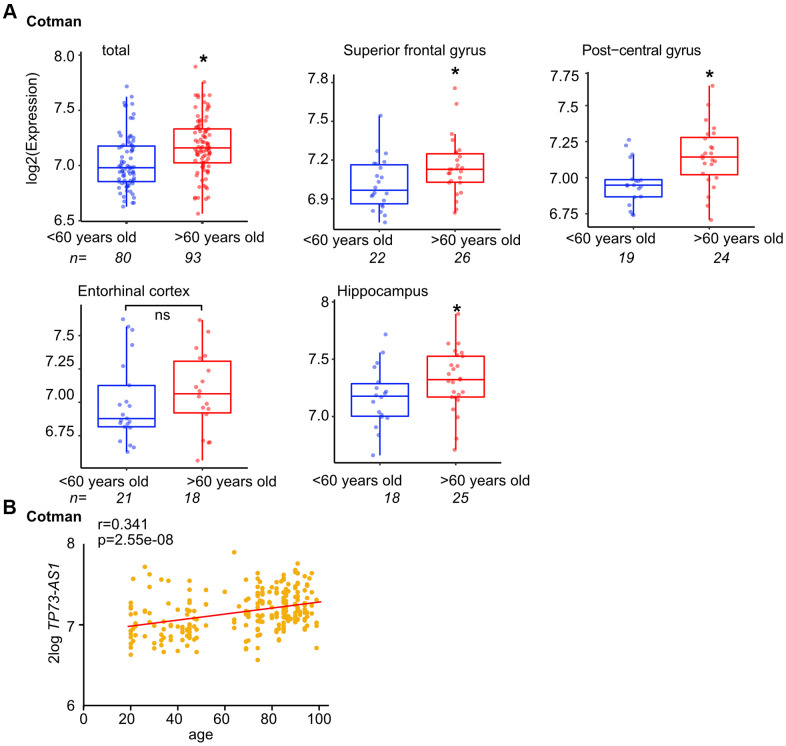
***TP73-AS1* is highly expressed in the aging brain.** (**A**) The levels of *TP73-AS1* in the old vs. young brain are shown. Data were obtained from the R2 website and the indicated dataset (GSE48350). (**B**) The correlation between the expression of *TP73-AS1* and age in the brain was determined using R2 and indicated dataset (GSE48350).

To learn more about how the expression of *TP73-AS1* is regulated, and given that its expression is increased during aging in glioma [33], we first asked if *TP73-AS1* expression is associated with natural aging in the brain and interrogated the Cotman dataset [42], using R2 software [45] and 60-year-old brains as a cutoff. We found that *TP73-AS1* is highly expressed in the aged brain and this is the case in most brain regions to which data is available (Figure 1A). In accord, the expression of *TP73-AS1* in the brain is correlated with aging (Figure 1B). Together, these data suggest that *TP73-AS1* is highly expressed in the aging brain.

**Figure 2:**
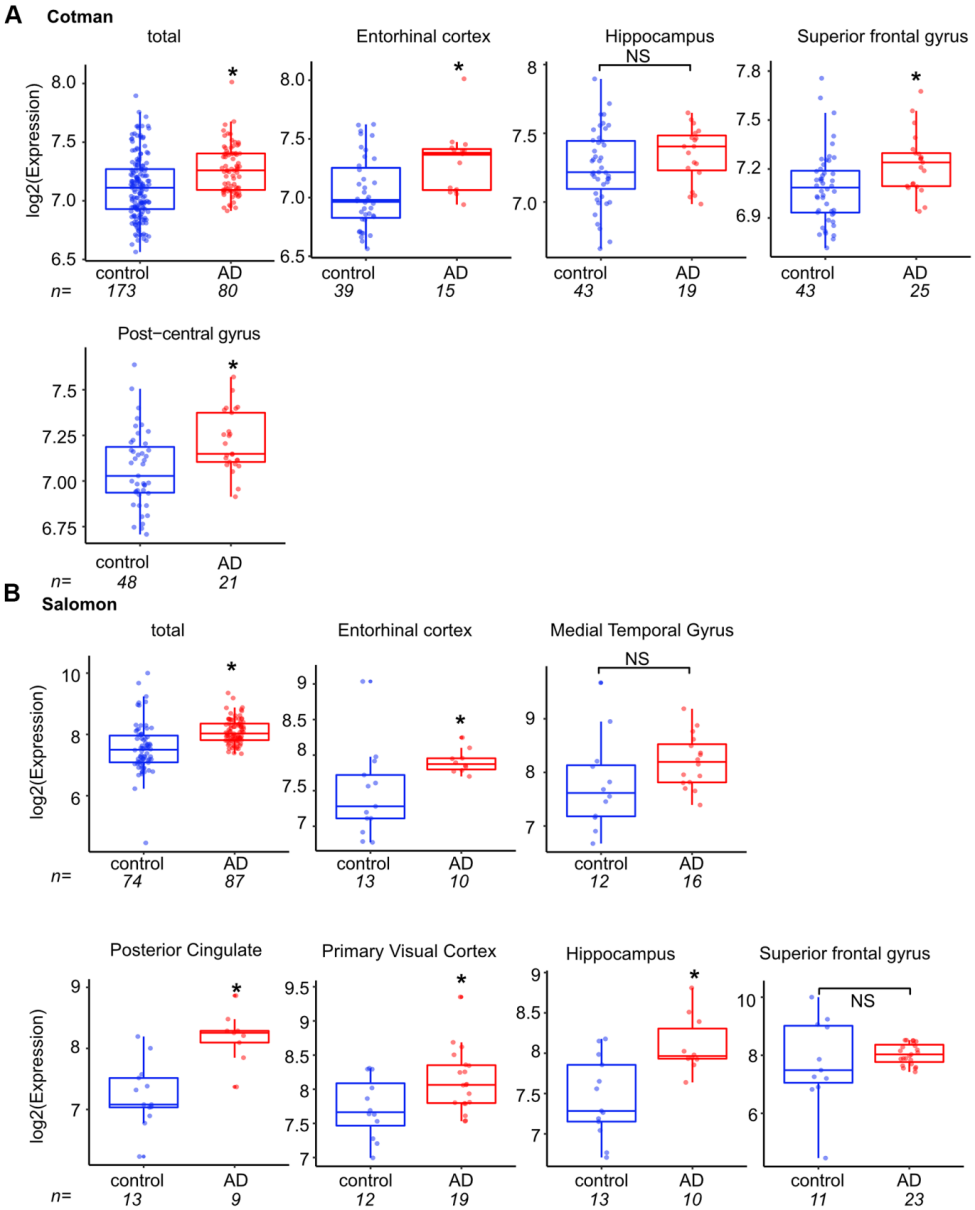
***TP73-AS1* is highly expressed in the pathological aging brain.** (**A**) The levels of *TP73-AS1* in Alzheimer’s (AD) vs. normal brain are shown. Data were obtained from R2 and the Cotman dataset (GSE48350). In both (**A**, **B**) panels, asterisks indicate statistically significant differences as calculated by the two-sample Wilcoxon test (*p < 0.05; NS, non-significant). (**B**) The levels of *TP73-AS1* in Alzheimer’s vs. normal brain are shown. Data were obtained from R2 and the Salomon dataset (GSE5281).

We next asked if *TP73-AS1* expression is also linked to pathological aging in the human brain. To this end, we interrogated the Cotman dataset and found that *TP73-AS1* is highly expressed in Alzheimer’s brain and that this is the case in most tested brain regions (Figure 2A). We confirmed these findings using a second dataset, the Salomon dataset [41] (Figure 2B) which supported our conclusion. While in the hippocampus (Cotman dataset), the median temporal and superior frontal gyrus (Salomon dataset) *TP73-AS1* expression was trending higher in Alzheimer’s vs. normal brain, these differences did not reach statistical significance, possibly due to a lower number of samples as compared with the total which was obtained by pooling of all samples from different brain regions.

The high expression *TP73-AS1* in the aging brain and our previous findings that *TP73-AS1* is highly expressed in GBM tumors, lead us to ask if the age of the patient is associated with *TP73-AS1* expression in GBM tumors. We analyzed *TP73-AS1* expression in GBM tumors of old and young patients using 60-year-old as a cutoff. Indeed, *TP73-AS1* is up-regulated in GBM tumors of older patients (Supplementary Figure 1A). In addition, we calculated the correlation between *TP73-AS1* expression and patient’s age and found that they are positively correlated (Supplementary Figure 1B).

Interestingly, we recently found that the TF YY1 and its gene targets are a prominent part of the pathological aging brain transcriptional program [38] and were intrigued as to whether YY1 is a *TP73-AS1* TF as well.

### *TP73-AS1* is induced by TMZ

**Figure 3:**
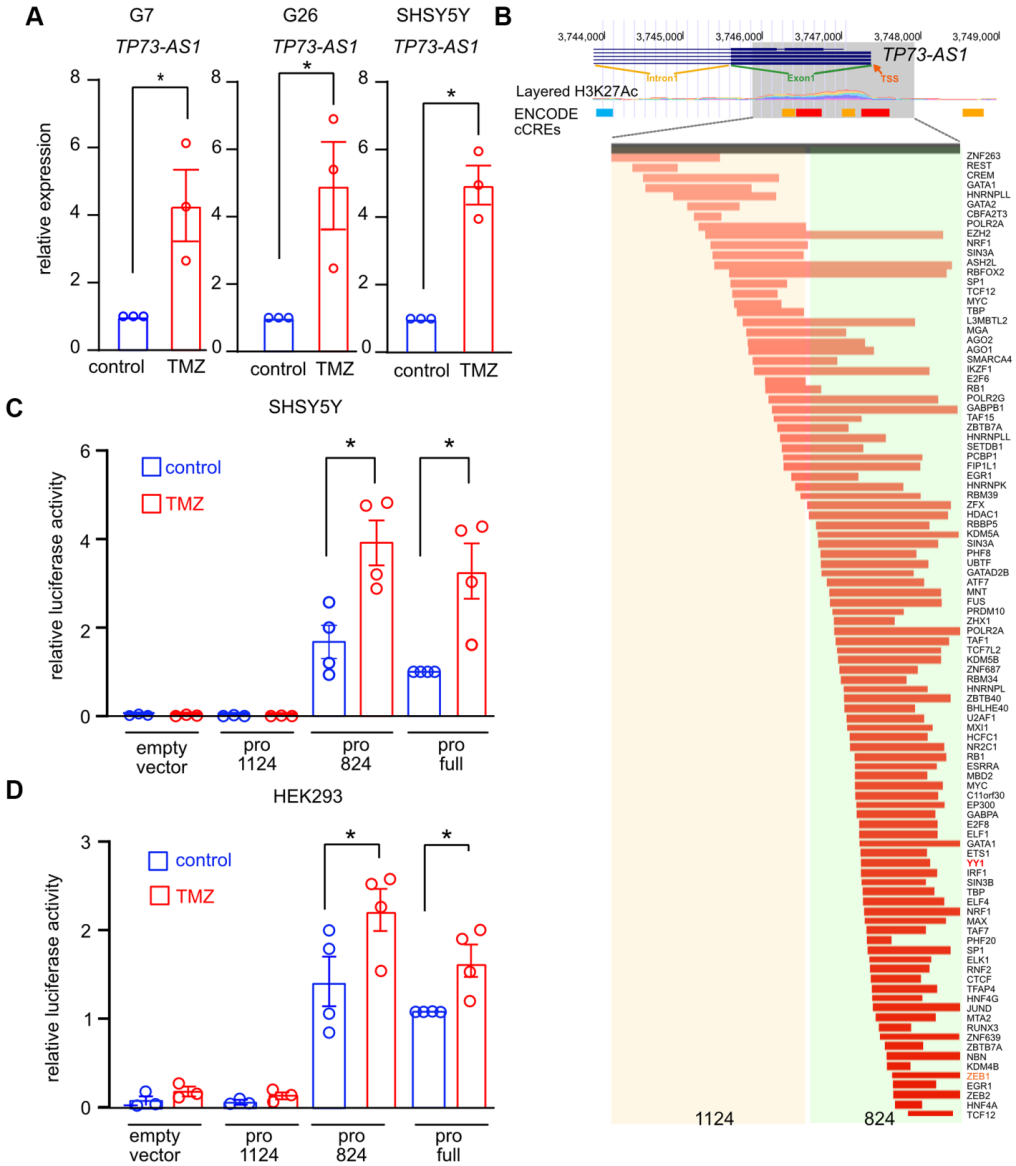
***TP73-AS1* is induced by TMZ.** (**A**) *TP73-AS1* levels in the indicated cell lines were measured using qRT-PCR. * p<0.05 in the two-tailed t-test. (**B**) The genomic region of the putative *TP73-AS1* promoter was identified using the UCSC browser. H3K27 acetylation and TF ChIP-SEQ data obtained from ENCODE are shown. Transcriptional start site (TSS), intron 1, and exon 1 are shown. The two promoter regions used in this study are highlighted. (**C**) Promoter activity assay was performed in the indicated cell lines using the dual luciferase assay. * p<0.05 in the two tailed t-test. (**D**) Promoter activity assay was performed in the indicated cell lines using the dual luciferase assay. * p<0.05 in the two tailed t-test.

Previously, we found that *TP73-AS1* protects GBM stem cells from TMZ, an alkylating agent, by promoting the expression of detoxifying genes such as *ALDH1A1* [31]*,* which neutralize aldehydes and other toxic molecules in the cell [46, 47]. We asked if *TP73-AS1* is induced by TMZ, as is expected from a gene promoting a detoxification transcriptional response. We treated two GBM cancer stem cell models, G7 and G26 [48], and a neuroblastoma model, SHY5Y, with TMZ (750 μM for 48 hours), after which we measured the expression of *TP73-AS1* using qRT-PCR (Figure 3A). We found that *TP73-AS1* is induced in TMZ treated cells.

We next asked if *TP73-AS1* expression is induced by a TF binding to its promoter. The sequence we defined as the promoter was chosen using ENCODE ChIP-seq data obtained using antibodies against histone K27 acetylation and specific TFs [49–52] (Figure 3B). To monitor promoter activity, we used the dual luciferase system. In short, the putative promoter sequence is cloned upstream to Firefly luciferase and cells are transfected with the vector harboring the promoter along with the transfection control vector encoding Renilla luciferase. Firefly and Renilla luciferase activity are measured, and the ratio between the two measurements reflects promoter activity. Transfected cells were treated with TMZ (750 μM for 48 hours) after which we measured Firefly/Renilla luciferase activity. We found that the full promoter is active in basal conditions and is further induced upon TMZ treatment (Figure 3C, 3D).

Based on the ENCODE TF binding data [49–52], we divided the promoter into two regions and cloned each region upstream to Firefly luciferase (Figure 3B). We transfected cells with each construct and measured Firefly and Renilla luciferase activity in basal and TMZ treated conditions (Figure 3C, 3D). We found that the 1124-promoter region was inactive in TMZ treated and non-treated cells, while the 824-promoter region was active in non-treated cells and induced upon TMZ treatment. Interestingly, YY1 was found to bind the 824-TP73-AS1 promoter region (ENCODE data; Figure 3B). We, therefore, concluded that *TP73-AS1* is a TMZ responsive gene and that the TF activating *TP73-AS1* upon TMZ treatment is most likely to be one of the TFs mapped to the 824-region.

### YY1 promotes the expression of *TP73-AS1* upon TMZ treatment

**Figure 4:**
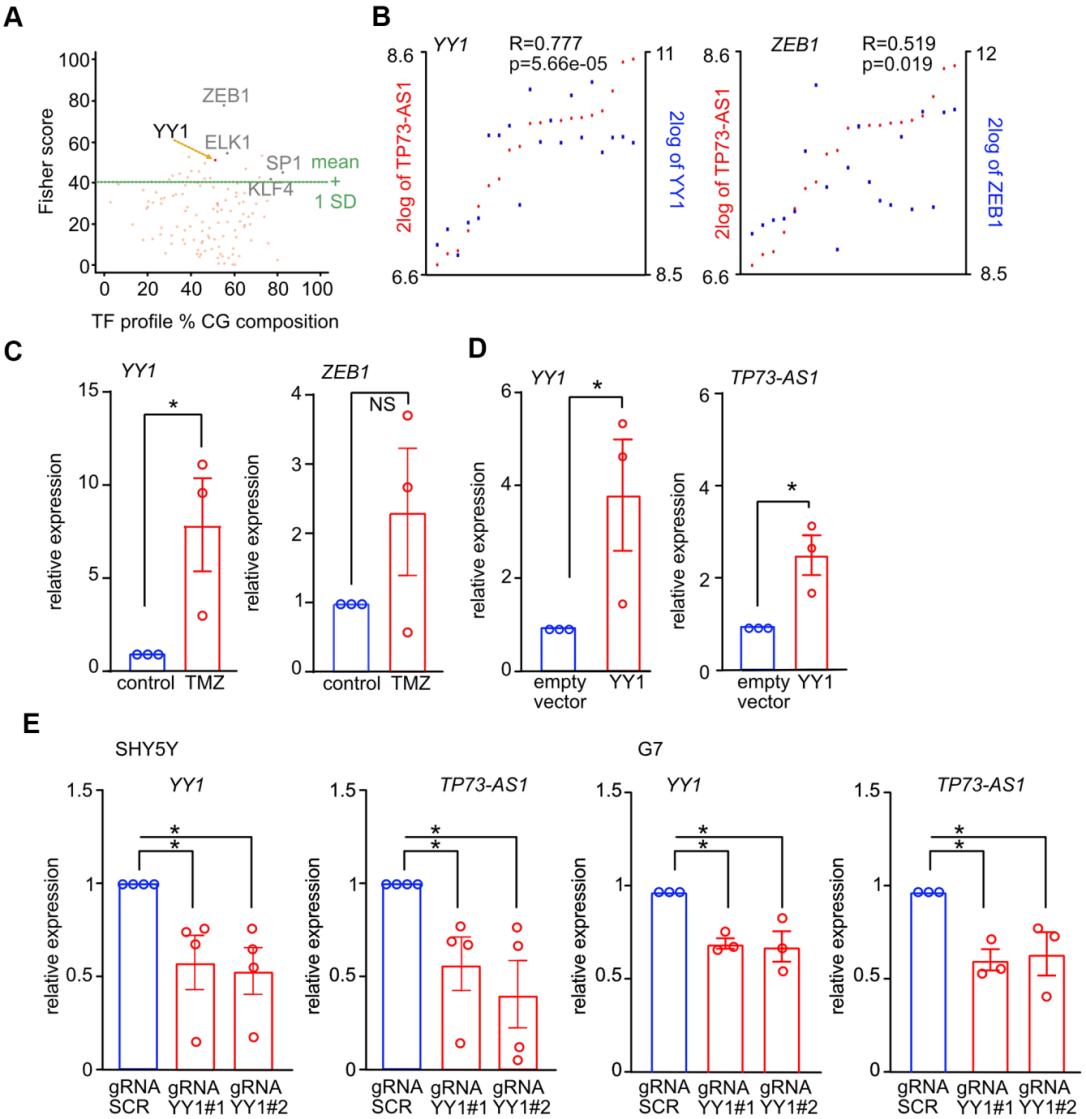
**YY1 promotes the expression of *TP73-AS1* upon TMZ treatment.** (**A**) TF binding sites statistically enriched in the list of genes up-regulated upon TMZ treatment in G7 cells were found using oPPOSUM (full list in Supplementary Table 1). Key TFs known to play a role in GBM are indicated. (**B**) Co-expression between the indicated TFs and *TP73-AS1* in GBM stem cells is shown. Data obtained using R2 and the Pollard dataset (GSE15209) [48]. (**C**) The levels of the indicated transcripts in SHY5Y cells treated or not with TMZ were measured using qRT-PCR. * p<0.05 in the two-tailed t-test; NS, not significant. (**D**) The levels of the indicated transcripts in SHY5Y cells transfected with the indicated constructs were measured using RT-qPCR. * p<0.05 in the two-tailed t-test; NS, not significant. (**E**) The indicated cell lines were engineered to express KREB-dCAS9 and gRNAs targeting *YY1*. Cells were treated with DOX for 10 days, to induce KREB-dCAS9. The levels of the indicated transcripts were measured using qRT-PCR. * p<0.05 in the two-tailed t-test; NS, not significant.

To identify the TF activating *TP73-AS1* upon TMZ treatment, we asked if there are specific TFs binding sites enriched in the list of genes upregulated during TMZ treatment, assuming that *TP73-AS1* is part of a TMZ transcriptional program whose genes may be regulated by a common TF. To this end, we analyzed the list of genes up regulated by TMZ in G7 GBM stem cells using data found in our previous publication [31] and oPPOSUM [53–55], and identified several TF consensus sites enriched in the promoters of these genes, including *YY1* and *ZEB1* (Figure 4A and Supplementary Table 1), both of which were found to be physically associated with the 824-promoter region (Figure 3B). Interestingly, both *YY1* and *ZEB1* are co-expressed with *TP73-AS1* in GBM stem cells (Figure 4B). This is not the case in bulk GBM tumor data (Supplementary Figure 2A), possibly due to *TP73-AS1* function in GBM stem cells, a sub-population of cells in bulk tumor [56]. Note, the r and p values are more significant in the case of *YY1*. To study the correlation of expression between *TP73-AS1* and *YY1* in normal aging we used GTEx data [43] and found a positive correlation in the different brain regions (Supplementary Figure 2B).

We measured *YY1* and *ZEB1* expression in TMZ treated and control cells and found that the expression of *YY1* but not *ZEB1* was significantly induced upon TMZ treatment (Figure 4C). Note, we tested if YY1 is also induced at the protein level and found that this is not the case (Supplementary Figure 3). We therefore conclude that the *YY1* gene responds to TMZ treatment and that YY1 putative function is not due to increased expression.

Having found that *YY1* responds to TMZ and that YY1 target genes are part of the aging brain transcriptional program [38], we hypothesized that YY1 plays a major role in promoting *TP73-AS1* expression upon TMZ treatment. We next asked if YY1 is involved in *TP73-AS1* up regulation upon TMZ treatment. We over expressed YY1 in SHSY5Y cells and measured *TP73-AS1* levels using RT-qPCR (Figure 4D). YY1 promoted *TP73-AS1* expression. Next, we used CRISPRi to down regulate *YY1* expression, with two independent gRNAs, and measured *TP73-AS1* levels using qRT-PCR in basal conditions (Figure 4E). We found that *YY1* depletion led to reduced *TP73-AS1* expression and therefore concluded that YY1 is a major *TP73-AS1* regulator. Indeed, the expression of *TP73-AS1* and *YY1* is correlated in both human pathological aging (AD) brain datasets (Supplementary Figure 2C).

In our previous work studying *TP73-AS1* in GBM or medulloblastoma, we asked if the expression of *TP73-AS1* and *p73* are correlated, as one would expect if they were to regulate each other in cis, and the answer was negative [31, 34]. To address the possibility that during aging *TP73-AS1* regulates *p73* (or vice versa), we performed a co-expression analysis and found that there is significant co-expression in one dataset (Salomon) but not the other (Cotman) (Supplementary Figure 2D). This discrepancy could be due to the higher number of samples in the former. Considering that the role of p73 in the aging brain is under debate [57], we decided to focus on YY1 as a putative regulator of *TP73-AS1*.

### YY1 directly promotes *TP73-AS1* expression and promoter activation

**Figure 5:**
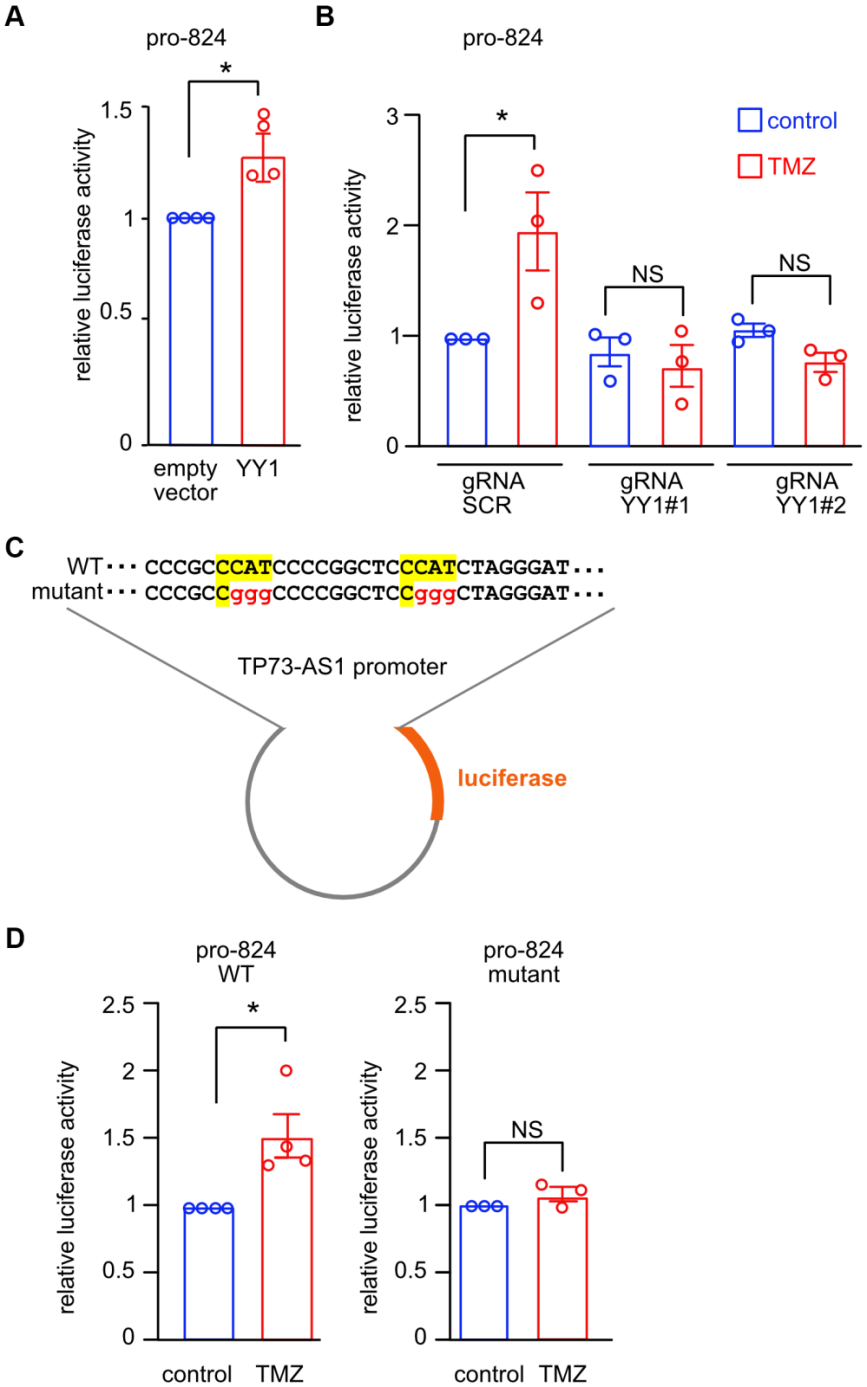
**YY1 directly promotes *TP73-AS1* promoter activation.** (**A**) The indicated promoter activity was measured in SHY5Y cells over expressing YY1 or empty vector. * p<0.05 in the two-tailed t-test; NS, not significant. (**B**) CRISPRi SHY5Y cells were treated with DOX for 10 days to induce *YY1* KD. Cells were treated with TMZ (750 μM for 48 hours). The activity of TP73-AS1-promoter-824 was measured using the dual luciferase assay. * p<0.05 in the two-tailed t-test; NS, not significant. (**C**) Schematic representation of the WT or mutant YY1 binding sites within the *TP73-AS1* promoter luciferase construct. (**D**) SHY5Y cells expressing WT or mutant promoter were treated with TMZ or DMSO control for 48 hours after which promoter activity was measured using dual luciferase assay. * p<0.05 in the two-tailed t-test; NS, not significant.

To investigate the interaction between YY1 and the promoter of *TP73-AS1*, we over expressed YY1 and measured the 824-promoter activity using luciferase activity assay (Figure 5A). We found that YY1 over expression increased 824-promoter activity. Importantly, CRISPRi knock down of YY1 prevented the induction of *TP73-AS1* upon TMZ treatment (Figure 5B).

We identified two canonical YY1 binding sites in the 824-promoter using Jaspar [31] and mutated the two binding sites to study their importance for promoter activation upon TMZ treatment (Figure 5C). We treated cells, expressing the wild type or mutant 824-promoter, with TMZ (750 μM for 48 hours), after which we measured Firefly/Renilla luciferase activity (Figure 5D). TMZ treatment led to increased promoter activation of the wild type but not the mutant 824-promoter. Together, these data strongly suggest that YY1 promotes *TP73-AS1* induction upon TMZ treatment by binding to and activating the 824-promoter region.

## DISCUSSION

GBM aggressiveness is tightly linked to temozolomide resistance [58]. The lncRNA *TP73-AS1* protects GBM stem cells from TMZ toxicity [31]. Previous studies of *TP73-AS1* expression in brain tumors focused on genetic and epigenetic mechanisms to explain its aberrant expression. In oligodendroglial tumors, reduced *TP73-AS1* expression was identified and correlated with a deletion of the p36.31–p36.32 region on chromosome 1, where *TP73-AS1* resides, and with *TP73-AS1* promoter hyper methylation [35]. In a study aimed at identifying GBM epigenetic subgroups, downregulation of *TP73-AS1* expression and hypermethylation of its promoter were identified as defining features of the IDH/G-CIMP+ subgroup [59]. Here, we found that YY1 promotes *TP73-AS1* expression and the activity of its promoter upon TMZ treatment, thus adding a mechanistic explanation to how *TP73-AS1* is induced in this context. Such a TF-lncRNA axis promoting TMZ resistance has been previously found. The lncRNA *MALAT1* is positively regulated by the TFs NF-kappaB and p53 which upon TMZ treatment, bind specific sequences within the *MALAT1* promoter and activate it, increasing *MALAT1* levels and, consequently, TMZ resistance [34].

Aging contributes to glioma incidence and aggressiveness and interestingly, the expression pattern of *TP73-AS1* is associated with key features linked to aging and glioma aggressiveness. These include high expression of *TP73-AS1* in EGFR amplified and IDH-wild type tumors [10]. These associations are in line with our findings that *TP73-AS1* expression correlates with aging and aggressiveness providing a possible molecular link explaining how aging contributes to GBM aggressiveness.

YY1 is a TF shown to carry out pro-tumorigenic functions [60] including promoting the expression of *c-MYC* by directly binding its promoter [61] or inhibiting the tumor suppressor activity of p53 upon DNA damage [62, 63]. YY1 was also shown to promote the expression of the stemness factor KLF4 [64] and has been implicated in promoting the expression of stemness factors in cancer stem cells [65]. Interestingly, the levels and activity of the transcription factor YY1 and its gene targets decline with age in T-cells [66] and increase with age in brain [38]. Moreover, higher mRNA levels of YY1 were found in Alzheimer diseased brain and YY1 was defined as a “master regulator” in Alzheimer disease [67]. Furthermore, in GBM stem cells, YY1 was shown to promote stemness, TMZ resistance and tumorigenicity, through yet unknown mechanisms [37].

Here we found that YY1 induces the expression of *TP73-AS1* upon TMZ treatment, thus providing a possible explanation for the protective functions of YY1, in light of the known function of *TP73-AS1* in promoting TMZ resistance in GBM stem cells [31]. Although *YY1* transcript level increase in response to TMZ treatment, and directly induces the expression of *TP73-AS1*, YY1 protein levels were not induced. Reasonable explanation for the upregulation at the mRNA levels but not the protein level, is that TMZ treatment may induce a cellular stress leading to inhibition of translation [68]. Nevertheless, YY1 is modified and regulated post-translationally. For example, YY1 is marked for degradation by SMURF2 E3 ubiquitin ligase [69], is tyrosine phosphorylated by the SRC kinases family [70] or subjected to acetylation and deacetylation [70]. Considering YY1 regulates multiple genes, the contribution of *TP73-AS1* to a given YY1 biological function is yet to be determined.

Aberrant YY1 function in aging brain was recently reported and attributed to reduced expression of its binding partner, SIRT6 [38]. Reduced SIRT6 expression, occurring during aging, leads to changes in the expression of genes which are regulated by YY1, many of which are involved in pathological aging. It is therefore possible that *TP73-AS1* is a YY1 target in aging brain.

In conclusion, we show that *TP73-AS1* levels increase in pathological and natural aging brain and upon TMZ treatment, and that YY1 directly activates the *TP73-AS1* promoter to induce its expression. These findings provide a plausible explanation for how the expression of *TP73-AS1* is regulated, and an interesting molecular link between aging and GBM.

## Supplementary Material

Supplementary Figures

Supplementary Table 1
