# Supplementary material for: TP73-AS1 is induced by YY1 during TMZ treatment and highly expressed in the aging brain
Source: Aging (Albany NY). 2021 Jun 11;13(11):14843–61. doi: 10.18632/aging.203182 (PMC8221307; doi:10.18632/aging.203182)
Supplement: Supplementary Figures [file aging-13-203182-s001.pdf]

SUPPLEMENTARY FIGURES

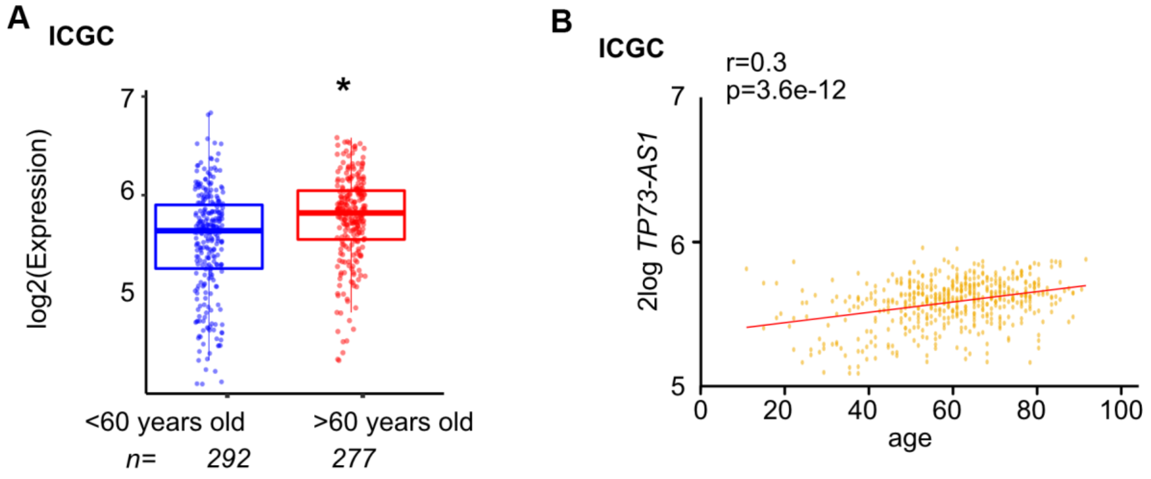

Supplementary Figure 1. TP73-AS1 is highly expressed in GBM tumors of aged patients. (**A**) The levels of *TP73-AS1* in GBM tumors obtained form the old vs. young patients are shown. Data were obtained from GBM-US donors (a total of 595 donors) from ICGC database [1]. (**B**) The correlation between the expression of *TP73-AS1* and age in the tumors of GBM patients. Data were obtained from GBM-US donors (a total of 595 donors) from ICGC database [1].

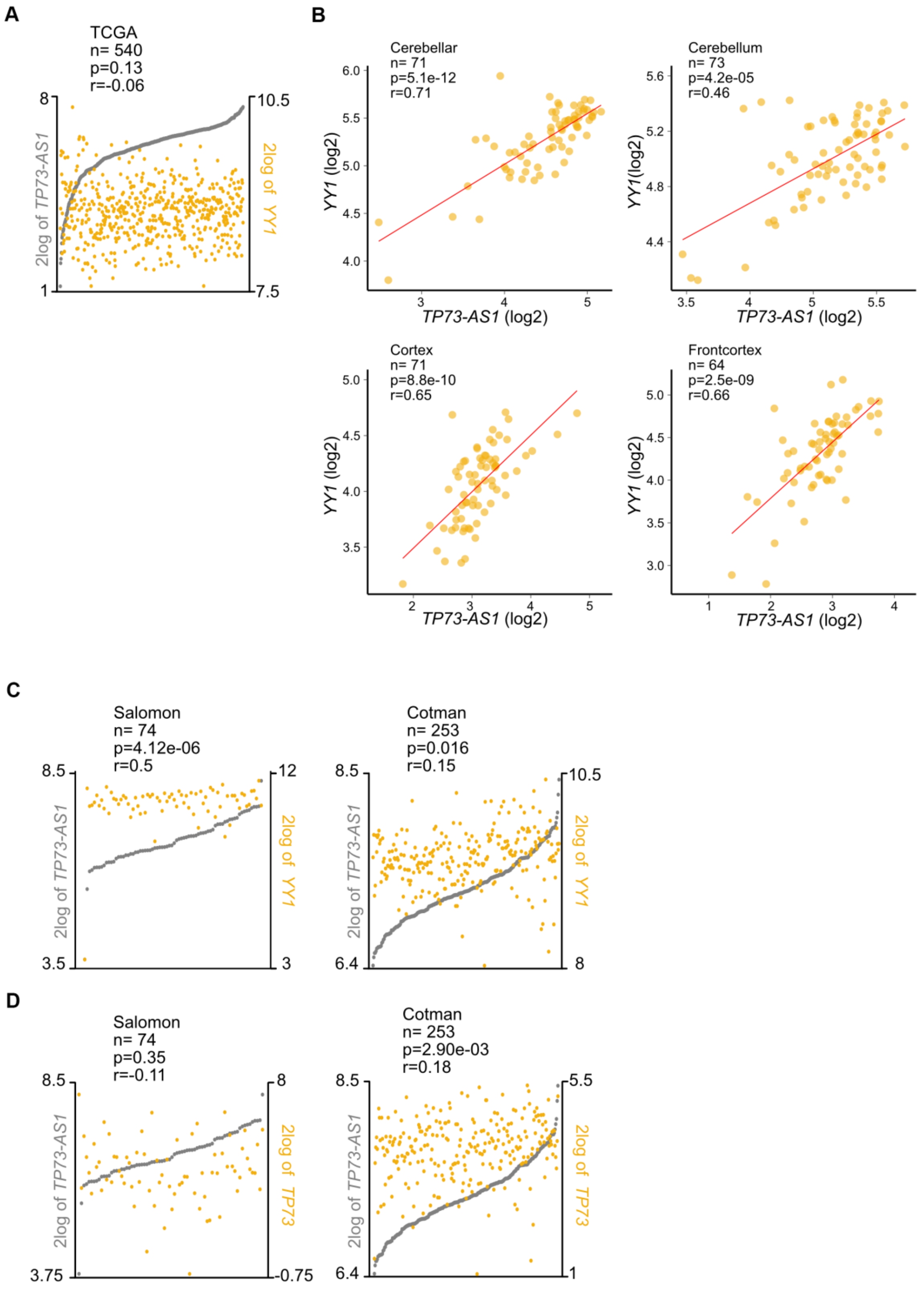

Supplementary Figure 2. YY1 and TP73-AS1 are co-expressed in GBM and aging brain. (**A**) The correlation between the expression of *TP73-AS1* and *YY1* in the GBM were determined using R2 and TCGA dataset. (**B**) The correlation between the expression of *TP73-AS1* and *YY1* across different brain parts in the GTEx dataset. (**C**) The correlation between the expression of *TP73-AS1* and *YY1* in aging brain were determined using R2 and the indicated datasets. (**D**) The correlation between the expression of *TP73-AS1* and *p73* in the aging were determined using R2 and indicated dataset.

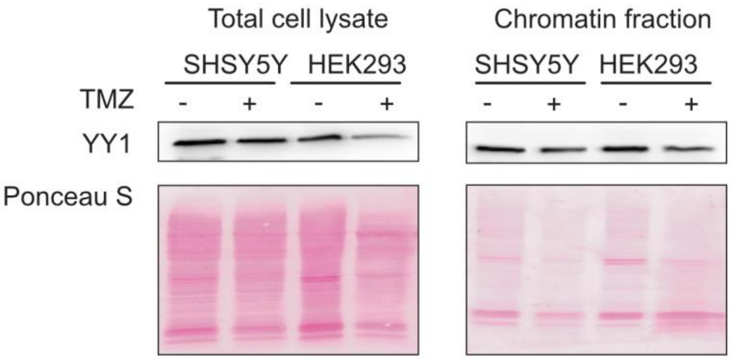

Supplementary Figure 3. TMZ does not induce YY1 at the protein level. The indicated cells treated or not with TMZ for 3 days after which the levels of YY1 in the total cell lysate or chromatin fraction were measured using western blot. Ponceau S was used to determine total protein loading.

REFERENCES

1. Zhang J, Bajari R, Andric D, Gerthoffert F, Lepsa A, Nahal-Bose H, Stein LD, Ferretti V. The International Cancer Genome Consortium Data Portal. Nat Biotechnol. 2019; 37:367–69. https://doi.org/10.1038/s41587-019-0055-9 PMID:30877282
